# Inflammatory Biomarkers Following Orthopedic Surgery: Current Evidence, Clinical Applications, and Future Perspectives—A Narrative Review

**DOI:** 10.3390/jcm15145399

**Published:** 2026-07-09

**Authors:** Anna Perek, Tomasz Reysner, Jowita Rosada-Kurasińska, Paweł Pietraszek, Justyna Marszałek-Buko, Bartłomiej Perek, Ewa Grelowska, Alicja Bartkowska-Śniatkowska, Małgorzata Reysner

**Affiliations:** 1 Department of Clinical Anesthesiology and Pain Management, Poznan University of Medical Sciences, 61-545 Poznań, Poland; 2 Department of Pediatric Anesthesiology and Intensive Care, Poznan University of Medical Sciences, 61-545 Poznań, Poland; 3 Department of Cardiac Surgery and Transplantology, Poznan University of Medical Sciences, 61-545 Poznań, Poland; 4 Department of Anesthesiology and Intensive Care, Poznan University of Medical Sciences, 61-545 Poznań, Poland

**Keywords:** orthopedic surgery, inflammatory biomarkers, CRP, IL-6, NLR, systemic immune-inflammation index, fracture healing, periprosthetic joint infection, perioperative monitoring

## Abstract

Orthopedic procedures trigger a complex inflammatory response that plays a central role in tissue repair and postoperative recovery. However, excessive or dysregulated inflammation may contribute to complications such as periprosthetic joint infection, thromboembolic events, delayed healing, or systemic organ dysfunction. Therefore, accurate perioperative monitoring of inflammatory activity has become increasingly important in orthopedic surgery. Evidence discussed in this review was identified through a literature search of PubMed, Scopus, and Web of Science databases covering publications from 2000 to 2026. **This** narrative review summarizes the current evidence regarding both traditional inflammatory biomarkers, including C-reactive protein (CRP), interleukin-6 (IL-6), tumor necrosis factor-α (TNF-α), procalcitonin (PCT), and D-dimers, as well as emerging biomarkers derived from complete blood count (CBC), such as the neutrophil-to-lymphocyte ratio (NLR), monocyte-to-lymphocyte ratio (MLR), platelet-to-lymphocyte ratio (PLR), systemic immune-inflammation index (SII), systemic inflammatory response index (SIRI), and aggregate index of systemic inflammation (AISI). **Particular** attention is devoted to the clinical utility of these biomarkers in orthopedic trauma, fracture healing, total joint arthroplasty, and the early detection of postoperative complications. Increasing evidence suggests that composite CBC-derived indices may provide a practical and cost-effective approach for perioperative risk stratification and prognosis assessment. Nevertheless, their interpretation remains challenging due to the lack of standardized cutoff values and the influence of multiple patient-related factors. **Current** evidence indicates that assessing biomarker kinetics and interpreting multiple inflammatory indices together may be more clinically valuable than isolated measurements. Future research should focus on standardization, validation in prospective studies, and integration of inflammatory biomarkers into personalized perioperative care pathways in orthopedic surgery.

## 1. Introduction

Every surgical intervention triggers a local and systemic inflammatory response that plays a fundamental role in tissue repair and postoperative recovery [1]. In orthopedic surgery, tissue disruption, bone injury, blood loss, and the implantation of foreign materials, such as prostheses, collectively activate both innate and adaptive immune pathways [1,2,3,4]. Although this response is physiological and necessary for healing, excessive or dysregulated inflammation may contribute to postoperative complications, including surgical site infection (SSI), periprosthetic joint infection (PJI), thromboembolic events, delayed recovery, acute organ dysfunction, and prolonged hospitalization [5,6,7,8].

The clinical relevance of perioperative inflammatory monitoring has become increasingly important due to the growing number of elderly and frail patients undergoing orthopedic procedures [6]. Advanced age, multimorbidity, impaired immune competence, and reduced physiological reserve make these individuals particularly vulnerable to adverse postoperative outcomes, including acute kidney injury, pulmonary complications, cardiovascular instability, and impaired rehabilitation [7,8]. Furthermore, increasing evidence suggests that optimized perioperative care, including effective analgesia, early mobilization, and multimodal rehabilitation strategies, may attenuate excessive inflammatory activation and improve postoperative recovery [9,10].

Traditionally, inflammatory monitoring in orthopedic surgery has relied on conventional biomarkers such as C-reactive protein (CRP), interleukin-6 (IL-6), tumor necrosis factor-α (TNF-α), procalcitonin (PCT), erythrocyte sedimentation rate (ESR), and D-dimers [11,12]. However, recent years have brought growing interest in inflammatory indices derived from routine complete blood count (CBC), including the neutrophil-to-lymphocyte ratio (NLR), monocyte-to-lymphocyte ratio (MLR), platelet-to-lymphocyte ratio (PLR), systemic immune-inflammation index (SII), systemic inflammatory response index (SIRI), and aggregate index of systemic inflammation (AISI) [13,14]. These biomarkers are inexpensive, widely available, and may provide additional insight into the dynamic interaction between innate immunity, adaptive immunity, and coagulation pathways during the perioperative period.

Despite increasing interest in inflammatory biomarkers, their interpretation in orthopedic surgery remains challenging. Physiological postoperative inflammation frequently overlaps with early manifestations of infection or other complications, while biomarker kinetics may be influenced by age, comorbidities, obesity, surgical invasiveness, blood loss, or perioperative management strategies. Moreover, the clinical significance and optimal cutoff values of novel CBC-derived indices remain insufficiently standardized [15,16,17,18,19].

Several reviews have previously examined selected inflammatory biomarkers in orthopedic surgery. However, most focused on individual biomarkers, specific surgical procedures, or isolated postoperative complications. In addition, recently developed complete blood count-derived inflammatory indices, including NLR, MLR, PLR, SII, SIRI, and AISI, have not been comprehensively discussed alongside traditional inflammatory markers within a single clinically oriented review.

The primary focus of this review is on systemic blood-based inflammatory biomarkers routinely used in orthopedic surgery, whereas local synovial biomarkers are discussed briefly in the context of periprosthetic joint infection.

## 2. Methods

This narrative review summarizes current evidence on inflammatory biomarkers used in orthopedic surgery and their potential clinical applications. A comprehensive literature search was performed using the PubMed, Scopus, and Web of Science databases. The search strategy included combinations of the following keywords and Medical Subject Headings (MeSH) terms: “orthopedic surgery”, “orthopaedic surgery”, “inflammatory biomarkers”, “C-reactive protein”, “interleukin-6”, “tumor necrosis factor-alpha”, “procalcitonin”, “D-dimer”, “neutrophil-to-lymphocyte ratio”, “platelet-to-lymphocyte ratio”, “systemic immune-inflammation index”, “systemic inflammatory response index”, “aggregate index of systemic inflammation”, “fracture healing”, “arthroplasty”, “periprosthetic joint infection”, and “postoperative complications”.

The literature search covered publications from January 2000 to May 2026. Additional relevant studies were identified through manual screening of reference lists of selected articles. A PRISMA-style flow summary was used to describe the literature selection process. The database search identified 2137 records: 612 from PubMed, 824 from Scopus, and 701 from Web of Science. An additional 42 records were identified through manual screening of reference lists. After removal of 548 duplicates, 1631 records were screened by title and abstract. Of these, 1337 records were excluded as not relevant to the aim of the review. A total of 294 full-text articles were assessed for eligibility. After full-text review, 130 articles were excluded because they were not directly related to orthopedic surgery (*n* = 32), had insufficient focus on inflammatory biomarkers (*n* = 28), were animal or in vitro studies without direct clinical relevance (*n* = 21), were conference abstracts or had unavailable full text (*n* = 17), were not available as full-text articles in English (*n* = 12), or contained overlapping data or had low relevance to the review aim (*n* = 20). Finally, 164 publications were included in the narrative synthesis (Supplementary Figure S1).

Articles were eligible for inclusion if they investigated inflammatory biomarkers in the context of orthopedic trauma, fracture healing, arthroplasty, perioperative monitoring, postoperative recovery, or orthopedic complications. Human clinical studies involving both adult and pediatric populations were considered. Review articles, observational studies, randomized controlled trials, and prospective or retrospective cohort studies were eligible for inclusion when they provided clinically relevant information regarding inflammatory biomarkers.

Studies were excluded if they were not directly related to orthopedic surgery, lacked sufficient clinical relevance, were published only as conference abstracts, or were not available as full-text articles in English. Animal and in vitro studies were not systematically reviewed; however, selected experimental studies were cited when necessary to provide mechanistic insights into inflammatory pathways and biomarker biology.

Two authors independently screened titles, abstracts, and full-text articles for relevance. Any discrepancies regarding study eligibility were resolved through discussion and consensus. Given the narrative nature of this review and the substantial methodological heterogeneity among the available studies, a formal quantitative synthesis or meta-analysis was not performed. Instead, evidence was synthesized narratively by biomarker type, clinical application, and orthopedic context.

Particular emphasis was placed on studies evaluating the clinical utility of inflammatory biomarkers and CBC-derived indices in risk stratification, postoperative monitoring, and complication prediction. Additional landmark studies describing the physiological and immunological mechanisms of surgical inflammation were included to provide a pathophysiological context.

A summary of selected clinically relevant studies evaluating inflammatory biomarkers following orthopedic procedures is presented in Table 1.

The available evidence was predominantly derived from retrospective observational studies, with fewer prospective studies, randomized controlled trials, and systematic reviews. Therefore, conclusions about the clinical applicability of inflammatory biomarkers should be interpreted with caution, particularly for emerging CBC-derived indices, for which validation studies remain limited [19,20,21,22,23,24,25,26].

The quality of evidence was assessed descriptively based on study design and methodological characteristics. Evidence on traditional biomarkers such as CRP, IL-6, and procalcitonin included prospective cohort studies, randomized studies, and systematic reviews [20,21]. In contrast, most studies evaluating CBC-derived inflammatory indices were retrospective observational investigations, often conducted at single centers with heterogeneous patient populations [19,22,23,24,25,26]. Consequently, the overall certainty of evidence was considered moderate for traditional biomarkers and low-to-moderate for emerging CBC-derived indices.

## 3. Physiological Response to Surgical Trauma

Surgical trauma initiates a complex neuroendocrine, metabolic, hemostatic, and inflammatory response collectively referred to as the surgical stress response [29,30,31]. The magnitude of this response depends primarily on the extent and duration of tissue injury, blood loss, surgical invasiveness, and patient-related factors such as age, frailty, immune competence, and pre-existing comorbidities [31,32].

**Figure 1:**
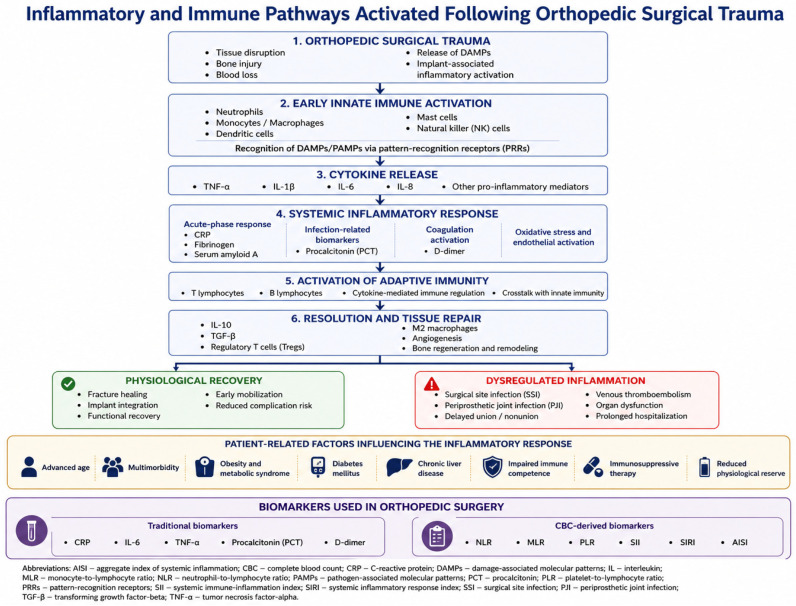
Schematic overview of the inflammatory and immune pathways activated following orthopedic surgical trauma. The figure summarizes the sequence of events from tissue injury and innate immune activation to cytokine release, systemic inflammatory response, adaptive immune regulation, tissue repair, and potential postoperative outcomes. Created by the authors using Canva (Canva Pty Ltd., Sydney, Australia).

In orthopedic surgery, disruption of skin, muscle, connective tissue, and bone integrity rapidly activates both innate and adaptive immune pathways [2]. Tissue injury and local bleeding trigger the recruitment of neutrophils, monocytes/macrophages, platelets, natural killer (NK) cells, and lymphocytes to the site of damage [33,34,35,36,37,38,39]. These cells participate not only in pathogen defense and removal of damaged tissue, but also in the initiation of tissue repair and bone healing processes [39,40]. Figure 1 summarizes the coordinated inflammatory and immune pathways activated following orthopedic surgical trauma.

The early inflammatory phase is characterized by the release of pro-inflammatory cytokines, including interleukin-1β (IL-1β), IL-6, IL-8, and tumor necrosis factor-α (TNF-α) [41,42]. Among them, IL-6 plays a central role in coordinating the systemic acute-phase response by stimulating hepatocytes to synthesize acute-phase proteins, including C-reactive protein (CRP), fibrinogen, serum amyloid A, haptoglobin, and D-dimers [43,44]. Simultaneously, IL-6, TNF-α, and IL-1β promote extra-thyroidal synthesis of procalcitonin (PCT), particularly during severe systemic inflammation and bacterial infection [45].

The kinetics of inflammatory mediators following surgery differ considerably and are clinically important. Cytokines such as TNF-α and IL-6 increase rapidly within hours after surgical trauma and decline relatively quickly, whereas acute-phase proteins such as CRP peak later, usually within 24–48 h [43,46,47]. Consequently, interpretation of postoperative inflammatory biomarkers should always consider both the timing of measurement and the expected physiological postoperative trajectory.

Importantly, postoperative inflammation is not exclusively detrimental. A controlled inflammatory response is essential for fracture healing, angiogenesis, tissue remodeling, and recovery after orthopedic interventions [48,49,50,51,52,53]. However, excessive or prolonged inflammatory activation may contribute to postoperative complications, including delayed healing, thromboembolic events, organ dysfunction, or surgical site infection [5,54].

Alongside pro-inflammatory activation, compensatory anti-inflammatory mechanisms are also initiated. Anti-inflammatory cytokines such as interleukin-10 (IL-10) and transforming growth factor-β (TGF-β) limit excessive immune activation and support tissue repair and remodeling [55]. Therefore, postoperative recovery depends largely on maintaining an appropriate balance between pro-inflammatory and anti-inflammatory pathways [54]. Even less invasive surgical techniques may induce measurable systemic inflammatory activation, although the magnitude of this response is generally related to the extent of tissue injury and procedural invasiveness [56,57].

## 4. Classic Biomarkers of the Inflammatory Response

Several classical inflammatory biomarkers have been widely used in perioperative medicine for decades, including CRP, IL-6, TNF-α, PCT, and D-dimers. These biomarkers reflect different phases and mechanisms of the inflammatory response and therefore exhibit distinct perioperative kinetics (Table 2). Consequently, they should be interpreted as complementary rather than interchangeable tools in postoperative monitoring following orthopedic procedures.

### 4.1. CRP

CRP is synthesized mainly by hepatocytes (but to a lesser extent also by smooth muscle cells, macrophages, endothelial cells, lymphocytes, and adipocytes) in response to infection (much more pronounced in the case of bacterial infection), nonspecific inflammation, or any tissue damage via IL-6 produced by macrophages or adipocytes [43,58,59].

CRP plays a key role not only in the first line of innate defense against primarily bacterial pathogens, but also participates in the removal of damaged and apoptotic cells [60].

Monitoring CRP is clinically significant in the diagnosis of early postoperative infections [61]. It may help distinguish a normal response to surgical trauma from complications [62]. This approach is most useful following high-risk or invasive procedures, such as major abdominal surgeries (for early detection of anastomotic leaks and infections), orthopedic procedures (particularly joint arthroplasty and spinal surgeries to identify deep surgical site infections), cardiothoracic surgeries (due to extensive iatrogenic injury), and emergency trauma procedures (due to the high risk of infectious complications) [63,64]. Peak CRP concentrations are usually observed within 24–48 h after surgery and may increase markedly above baseline values, depending on the extent of surgical trauma [46]. Therefore, a persistently high level or a secondary rise after the 3rd day suggests infection rather than physiological inflammation caused by trauma. Consequently, CRP assessment is most useful between the 3rd and 7 days after invasive procedures. Monitoring CRP also has clinical significance for assessing the efficacy of antibiotic therapy [65]. One of the main limitations of CRP is its relatively low specificity, as elevated levels may occur in virtually all inflammatory states, including infection, trauma, autoimmune disorders, and malignancy [58,59,60,66]. Therefore, postoperative CRP interpretation should always consider perioperative kinetics and the overall clinical context.

### 4.2. IL-6

IL-6 is a pleiotropic cytokine produced by monocytes/macrophages, neutrophils, lymphocytes, endothelial cells, fibroblasts, and keratinocytes in response to tissue injury, inflammation, or infection [67]. In the perioperative setting, monocytes/macrophages and other innate immune cells are considered the major source of IL-6 release following surgical trauma [42,67]. IL-6 plays a central role in the early inflammatory response by stimulating acute-phase protein synthesis in hepatocytes, particularly the production of CRP and fibrinogen [43,67]. Due to its rapid kinetics, IL-6 concentrations typically increase within hours after surgery and peak earlier than CRP, often within the first 6–24 h [20,47]. Therefore, IL-6 is considered one of the earliest biomarkers of postoperative inflammation. Clinically, IL-6 may be useful for early detection of severe systemic inflammatory response, cytokine release syndrome, postoperative infection, or sepsis [47]. Compared with CRP or white blood cell count, IL-6 may provide earlier information regarding excessive inflammatory activation and treatment response [20]. However, despite its high sensitivity, routine clinical application remains limited by its short half-life, interpatient variability, and limited availability in everyday perioperative practice.

### 4.3. TNF-α

TNF-α is an early pro-inflammatory cytokine produced mainly by macrophages/monocytes, T lymphocytes, NK cells, neutrophils, and dendritic cells in response to tissue injury, infection, or physiological stress [68,69]. Following surgical trauma, TNF-α contributes to activation of the cytokine cascade, leukocyte recruitment, endothelial activation, and amplification of the acute inflammatory response [70].

As an early-response cytokine, TNF-α concentrations increase rapidly after injury, usually peaking within 30–120 min [71]. Due to its very short half-life, systemic concentrations decline quickly unless severe inflammation or infection persists [72].

Although TNF-α plays an important pathophysiological role in acute inflammation, its clinical utility as a routine perioperative biomarker remains limited. Rapid kinetics, short half-life, and relatively low availability reduce its practical usefulness in postoperative monitoring compared with CRP or IL-6.

Beyond its role in acute inflammation, TNF-α also participates in bone remodeling and fracture healing through regulation of osteoclast and osteoblast activity. Experimental and clinical evidence suggests that excessive TNF-α signaling may contribute to impaired bone regeneration and chronic inflammatory bone loss. Therefore, TNF-α may serve not only as a biomarker of inflammation but also as a mechanistic mediator influencing orthopedic outcomes [48,50,53,72].

### 4.4. PCT

In healthy individuals, PCT is rapidly converted to calcitonin (CT) after its release from thyroid C cells; therefore, under normal conditions, its concentration in the blood is extremely low [73]. Following trauma (including surgical trauma) or sepsis, it is also produced by other cells [74]. The strongest stimulators are bacterial toxins (e.g., LPS) and pro-inflammatory cytokines such as TNF-α, IL-6, and IL-1 [75]. Interestingly, interferon gamma (IFN-γ), produced during viral infections, inhibits PCT synthesis, making it highly specific to bacterial pathogens [76]. PCT levels may also be elevated in cases of severe trauma, serious burns, and in the postoperative phase, even in the absence of bacterial infection [77]. Induced by inflammatory cytokines and bacterial endotoxins, it rises within 4–12 h, peaks after 12–24 h, and has a half-life of 20–24 h [78]. A normal host immune response, supported by prophylactic antibiotics, typically reduces PCT levels by 50% within 24 h [79].

PCT is crucial for the diagnosis of sepsis, the rational use of antibiotics, and the monitoring of therapy in intensive care units [79,80]. Furthermore, no correlation between PCT and calcitonin was observed in patients with infection [81]. The clinical application of PCT includes the diagnosis of sepsis due to its high specificity in distinguishing bacterial sepsis from other types of shock or systemic inflammation, as well as support in selecting the most appropriate antibiotic regimen, particularly the optimal timing for discontinuation. The latter reduces unnecessary antimicrobial use [74].

The main limitation appears to be the use of PCT levels to monitor local infections. However, clinical evidence is conflicting. In some studies, PCT measurements were effective, but in others, they failed to distinguish between infected and uninfected peripheral tissue injury [82,83,84]. In orthopedic surgery, the interpretation of PCT requires caution because postoperative elevations may also occur after major surgical trauma in the absence of infection [76]. Furthermore, its diagnostic value appears to be greater in systemic bacterial infections and sepsis than in localized orthopedic infections [81,82,84].

### 4.5. D-Dimer

D-dimer is an inactive, soluble product of fibrin degradation that is ultimately cleared from the blood by the kidneys and the reticuloendothelial system [85]. It is used to rule out deep vein thrombosis (DVT) and pulmonary embolism (PE) in patients with low or moderate pre-test probability [86]. It is also used to assess the risk of VTE recurrence and to determine the duration of anticoagulant therapy [87]. D-dimer levels rise consistently after surgery (beginning approximately 2 h post-surgery) due to iatrogenic trauma and activation of the coagulation cascade, typically peaking (often at levels twice as high as preoperative levels) between the 1st and 7th day (depending on the invasiveness and duration of the surgery), followed by a slow decline [88]. Generally speaking, for less invasive orthopedic surgeries, the peak is reached within 1–2 days; for spinal surgeries, within 5 days; and for extensive abdominal surgeries, even within 7 days [89]. Although levels often remain elevated for up to 3–4 weeks, a significant, nonspecific increase is a normal phenomenon, making them less reliable for diagnosing VTE in this early postoperative period. In orthopedic patients, interpretation of postoperative D-dimer levels is particularly challenging because surgical trauma itself markedly activates coagulation and fibrinolysis pathways [90]. Therefore, elevated postoperative D-dimer concentrations should be interpreted cautiously and always in conjunction with clinical assessment and imaging studies when thromboembolic complications are suspected.

The typical perioperative kinetics and clinical interpretation of classical inflammatory biomarkers are summarized in Table 3.

Although the perioperative kinetics of classical biomarkers are relatively well described, universal cut-off values remain difficult to define because they vary according to the type and extent of surgery, patient age, comorbidities, baseline inflammatory status, and timing of measurement. Therefore, serial postoperative trends and secondary increases may be more clinically informative than isolated single values.

### 4.6. Erythrocyte Sedimentation Rate (ESR)

Erythrocyte sedimentation rate (ESR) is one of the oldest laboratory markers of systemic inflammation and remains widely used in orthopedic practice. ESR reflects the tendency of erythrocytes to aggregate under the influence of acute-phase proteins, particularly fibrinogen. Unlike CRP and IL-6, ESR demonstrates relatively slow kinetics and may remain elevated for several weeks following surgery.

In orthopedic surgery, ESR is most commonly used as an adjunctive biomarker in the diagnosis of periprosthetic joint infection (PJI) and chronic inflammatory complications. Elevated ESR values may support suspicion of infection when interpreted together with CRP, microbiological findings, imaging studies, and clinical assessment. However, ESR lacks specificity and may be influenced by advanced age, anemia, autoimmune diseases, malignancy, and chronic inflammatory disorders.

Due to its prolonged normalization time, ESR is generally less useful than CRP for monitoring early postoperative inflammatory changes. Nevertheless, its widespread availability, low cost, and incorporation into several diagnostic algorithms for PJI continue to support its clinical relevance in orthopedic practice [11,60,91,92].

## 5. Inflammatory Biomarkers Derived from Complete Blood Count

In recent years, increasing attention has been directed toward inflammatory biomarkers derived from routine CBC, including simple ratios such as NLR, MLR, PLR, as well as composite indices including SII, SIRI, and AISI [93,94].

These biomarkers are inexpensive, rapidly available, and routinely obtained in everyday perioperative practice. Unlike isolated inflammatory markers, CBC-derived indices may better reflect the dynamic interactions among innate immunity, adaptive immunity, platelet activation, and the physiological stress response following surgical trauma [95]. CBC-derived inflammatory indices were investigated long before the COVID-19 pandemic; however, the pandemic substantially accelerated interest in their prognostic value and promoted broader clinical evaluation across multiple medical specialties [96]. Physiologically, surgical trauma is associated with rapid neutrophil and platelet activation, accompanied by transient lymphopenia related to neuroendocrine stress response and cortisol release [97]. Consequently, inflammatory indices derived from CBC may reflect the balance between pro-inflammatory activation and immune regulation during the perioperative period.

### 5.1. Simple Morphological Indices

Among the most commonly studied CBC-derived biomarkers are NLR, MLR, and PLR [21,98]. These indices are considered surrogate markers of the balance between systemic inflammation and immune regulation.

The NLR reflects the relationship between innate inflammatory activation (neutrophilia) and adaptive immune suppression (lymphopenia) [99]. Following surgical trauma, increased neutrophil counts combined with postoperative lymphocyte reduction contribute to elevated NLR values. Due to its rapid dynamics, NLR has been increasingly investigated as an early perioperative inflammatory biomarker.

Elevated NLR values have been associated with increased postoperative complications, infectious events, thromboembolic complications, prolonged hospitalization, and poorer outcomes in various surgical and medical conditions [99]. In orthopedic surgery, NLR appears particularly promising due to its rapid postoperative kinetics and widespread availability.

However, NLR remains a highly non-specific biomarker and may be influenced by advanced age, smoking, obesity, chronic inflammatory diseases, steroid therapy, malignancy, and perioperative physiological stress [100,101,102]. Therefore, isolated NLR values should always be interpreted cautiously and in conjunction with clinical findings and other laboratory markers.

The MLR reflects the relationship between monocyte-mediated inflammatory activation and lymphocyte-dependent immune regulation [103]. Increased MLR values have been associated with chronic inflammation, postoperative complications, and poorer prognosis in several clinical settings [104,105,106]. Compared with NLR, MLR has been less extensively investigated in orthopedic surgery, although preliminary evidence suggests potential utility in postoperative risk assessment and infection monitoring.

The PLR combines information regarding inflammatory activation and platelet-related coagulation response [104]. Elevated PLR values may reflect increased inflammatory activity, platelet activation, and a prothrombotic state. In orthopedic patients, PLR has been investigated mainly as a potential predictor of postoperative complications, thromboembolic events, and infection risk [107].

Overall, simple CBC-derived inflammatory indices are attractive due to their low cost and universal availability. However, reported threshold values vary considerably between studies and orthopedic populations. Therefore, dynamic perioperative changes and serial measurements may be more clinically informative than isolated values.

### 5.2. Composite Markers Derived from Complete Blood Count

More recently, composite inflammatory indices such as SII, SIRI, and AISI have been proposed as more comprehensive biomarkers of systemic inflammation [108]. By combining neutrophil, lymphocyte, monocyte, and platelet counts, these indices may better reflect interactions among inflammation, immune dysregulation, and coagulation pathways.

#### 5.2.1. SII and SIRI

Initially investigated mainly in oncology, SII and SIRI have subsequently been evaluated in cardiovascular, autoimmune, infectious, and perioperative conditions [108,109]. These indices may provide a broader assessment of systemic inflammatory status than isolated blood cell ratios alone. In orthopedic surgery, elevated SII and SIRI values have been associated with increased postoperative complications, infection risk, thromboembolic events, and poorer functional outcomes [23,24,110]. Their potential clinical value appears particularly relevant in elderly and frail patients undergoing major orthopedic procedures. Despite promising preliminary findings, current evidence remains heterogeneous and is predominantly limited to retrospective observational studies. Furthermore, optimal cutoff values remain poorly standardized across studies and patient populations. Representative study-specific cut-off values reported in orthopedic populations are summarized in Supplementary Table S1.

#### 5.2.2. AISI

AISI combines neutrophil, monocyte, platelet, and lymphocyte counts into a single composite marker that reflects the global inflammatory and immune status [111]. Compared with simpler indices, AISI may theoretically provide a more comprehensive representation of perioperative inflammatory activation. Recent studies suggest that elevated AISI values may correlate with poorer postoperative outcomes, increased complication rates, and higher mortality risk in various inflammatory conditions [112,113]. In orthopedic surgery, evidence regarding AISI remains limited but promising, particularly for infection prediction and postoperative risk stratification [114]. Nevertheless, the clinical interpretation of AISI remains challenging due to significant inter-study variability, lack of standardized thresholds, and strong influence of patient-related confounding factors.

Despite growing interest in CBC-derived inflammatory biomarkers, several important limitations warrant emphasis. Most of the currently available evidence comes from retrospective single-center studies with heterogeneous patient populations and inconsistent cutoff values [14,15,16]. Moreover, these biomarkers are highly susceptible to confounding factors such as advanced age, obesity, smoking, diabetes, chronic inflammatory disorders, malignancy, steroid therapy, chemotherapy, and perioperative stress response [115,116,117]. Consequently, CBC-derived indices should currently be considered supportive rather than definitive diagnostic tools, and their interpretation should always be integrated with clinical assessment and conventional laboratory findings.

Future studies should focus on prospective validation, standardization of cutoff values, and evaluation of dynamic perioperative changes rather than isolated measurements.

The major advantages and limitations of CBC-derived inflammatory biomarkers are summarized in Table 4.

Importantly, currently available studies use heterogeneous threshold values and different measurement time points; therefore, no universally accepted cutoff values can presently be recommended for routine orthopedic practice.

## 6. Clinical Application of Inflammatory Biomarkers Following Orthopedic Procedures

Inflammatory biomarkers are increasingly used in orthopedic surgery not only for the detection of postoperative complications, but also for perioperative risk stratification, monitoring of recovery, and prediction of clinical outcomes. Their interpretation is particularly important in elderly and frail patients, in whom excessive inflammatory activation may contribute to poorer postoperative recovery, delayed mobilization, prolonged hospitalization, and increased mortality risk [14,120,121,122].

Among orthopedic applications, the greatest clinical interest currently centers on the early detection of periprosthetic joint infection (PJI), surgical site infection (SSI), thromboembolic complications, and impaired fracture healing. However, interpretation of postoperative inflammatory biomarkers remains challenging because physiological postoperative inflammation frequently overlaps with early pathological inflammatory processes [121,123,124,125].

### 6.1. Total Joint Arthroplasty

Total hip and knee arthroplasty are associated with substantial perioperative inflammatory activation resulting from extensive tissue injury, bone resection, blood loss, and implantation of foreign material [14,125,126,127]. Although postoperative elevation of inflammatory biomarkers is expected, excessive or prolonged increases may indicate postoperative complications, particularly PJI or SSI.

CRP remains one of the most commonly used biomarkers after arthroplasty due to its availability and relatively predictable postoperative kinetics [128,129,130]. Physiologically, CRP levels typically peak within 48 h after surgery and gradually decline thereafter. Persistent elevation or secondary postoperative increase may raise suspicion for infection or ongoing inflammatory complications [131,132,133].

Similarly, IL-6 shows rapid postoperative elevation and may provide earlier information on excessive inflammatory activation than CRP [132,134]. However, due to short half-life and limited routine availability, IL-6 remains primarily a complementary biomarker in perioperative monitoring.

Growing evidence suggests that CBC-derived inflammatory indices, such as NLR, PLR, SII, and SIRI, may provide additional prognostic information following arthroplasty [22,118,135,136]. Elevated perioperative NLR and SII values have been associated with increased postoperative complications, prolonged hospitalization, delayed recovery, and higher infection risk.

Importantly, several studies suggest that dynamic perioperative trends may be more clinically informative than isolated biomarker measurements. Failure of CRP normalization, secondary elevation of CRP, or persistently elevated NLR and SII values may indicate abnormal postoperative recovery and warrant further diagnostic evaluation [23,119,137,138].

### 6.2. Orthopedic Trauma and Fracture Healing

Inflammation plays a crucial role in fracture healing and bone regeneration. Following traumatic injury, activation of inflammatory pathways initiates recruitment of immune cells, angiogenesis, and tissue remodeling processes necessary for successful bone repair [48,49,139].

The early inflammatory phase following fracture is characterized by increased production of IL-1β, IL-6, TNF-α, and other mediators involved in hematoma formation and recruitment of reparative cells [48,140,141]. Controlled inflammatory activation is necessary for physiological healing; however, excessive systemic inflammation may impair bone regeneration and increase the risk of delayed union, infection, or systemic complications [142,143].

Recent evidence suggests that CBC-derived biomarkers such as NLR, SII, and SIRI may help identify trauma patients at increased risk of complications and poorer functional recovery [25,26,144]. Elevated inflammatory indices have also been associated with increased mortality risk and prolonged hospitalization in elderly trauma patients.

Nevertheless, currently available evidence remains limited predominantly to retrospective observational studies with heterogeneous patient populations and inconsistent cutoff values. Therefore, further prospective validation studies are required before routine clinical implementation.

### 6.3. Periprosthetic Joint Infection and Surgical Site Infection

Early diagnosis of periprosthetic joint infection (PJI) remains one of the greatest challenges in contemporary orthopedic surgery. Interpretation of inflammatory biomarkers in this setting is particularly difficult because physiological postoperative inflammation frequently overlaps with early manifestations of infection [91,145,146].

CRP and ESR remain the most commonly used laboratory biomarkers in routine clinical practice [11,128,147]. However, their specificity is limited in the immediate postoperative period. Consequently, increasing attention has been paid to CBC-derived inflammatory indices, including NLR, PLR, SII, and SIRI, as potential adjunctive biomarkers for the early detection of infection [19,119,148].

Several studies have demonstrated that persistently elevated or secondarily increased inflammatory indices may be associated with a higher risk of PJI and SSI [119,146,148,149]. Furthermore, the combined interpretation of conventional inflammatory markers and CBC-derived indices may improve postoperative diagnostic accuracy.

Nevertheless, no single biomarker currently demonstrates sufficient sensitivity and specificity to independently confirm or exclude infection. Therefore, inflammatory biomarkers should always be interpreted in conjunction with clinical examination, microbiological findings, and imaging studies [19,92].

#### Local Biomarkers for Periprosthetic Joint Infection

Although serum inflammatory biomarkers remain useful for postoperative monitoring, increasing attention has been directed toward local synovial biomarkers for the diagnosis of periprosthetic joint infection (PJI). Because systemic inflammatory markers may be influenced by surgical trauma and comorbidities, analysis of synovial fluid can provide more direct information regarding the local inflammatory environment surrounding the prosthesis [91,92].

Among currently available biomarkers, alpha-defensin has emerged as one of the most extensively studied diagnostic tools. Produced by activated neutrophils in response to bacterial pathogens, alpha-defensin demonstrates high diagnostic accuracy and is relatively unaffected by prior antibiotic administration. Consequently, it has been incorporated into several contemporary diagnostic algorithms for PJI [91,92].

Calprotectin, a protein released by activated neutrophils and monocytes, has recently gained considerable attention as a rapid and cost-effective biomarker of joint inflammation. Several studies have reported promising sensitivity and specificity for detecting PJI, suggesting that calprotectin may become an attractive adjunctive diagnostic tool in routine clinical practice [122].

Additional synovial biomarkers include leukocyte esterase, synovial leukocyte count, polymorphonuclear neutrophil percentage (PMN%), and synovial interleukin-6 (IL-6). These markers may improve diagnostic accuracy when interpreted together with microbiological findings, imaging studies, and serum inflammatory biomarkers [92,134].

Current evidence suggests that no single biomarker is sufficient to independently confirm or exclude PJI. Therefore, a multimodal diagnostic approach combining clinical assessment, serum biomarkers, synovial fluid analysis, microbiological testing, and imaging remains the preferred strategy for accurate diagnosis [91,92].

### 6.4. Thromboembolic Complications

Major orthopedic procedures are associated with substantial activation of coagulation and fibrinolytic pathways, increasing the risk of venous thromboembolism (VTE) [150]. Inflammatory biomarkers such as D-dimers, PLR, SII, and NLR have been investigated as potential predictors of thromboembolic complications following orthopedic surgery [22,114,151].

However, interpretation of these biomarkers remains difficult because postoperative inflammation itself strongly influences coagulation activation. Consequently, elevated inflammatory indices should not be considered diagnostic of VTE in isolation and should always be accompanied by clinical correlation and imaging confirmation.

D-dimer concentrations increase physiologically after major orthopedic procedures due to activation of coagulation and fibrinolytic pathways. Consequently, interpretation of postoperative D-dimer levels requires consideration of expected postoperative kinetics and clinical context. Persistently increasing values, particularly when accompanied by clinical symptoms, may warrant further diagnostic evaluation for venous thromboembolism [86,88,89,90,150,151].

Although D-dimer levels are frequently elevated after major orthopedic procedures, their interpretation remains challenging because postoperative coagulation activation may persist for several weeks in uncomplicated recovery. Therefore, isolated D-dimer measurements should be interpreted with caution and, when venous thromboembolism is suspected, preferably combined with clinical assessment and imaging studies.

### 6.5. Limitations of Inflammatory Biomarkers in Orthopedic Surgery

Despite increasing interest in inflammatory biomarkers, several important limitations restrict their routine clinical application in orthopedic surgery. First, physiological postoperative inflammation substantially overlaps with pathological inflammatory responses, particularly during the early postoperative period [152,153]. Second, most currently available cutoff values remain poorly standardized and vary significantly between studies and patient populations [13,154].

Moreover, inflammatory biomarkers may be strongly influenced by advanced age, obesity, smoking, chronic inflammatory diseases, malignancy, corticosteroid therapy, perioperative blood transfusions, surgical invasiveness, and postoperative complications unrelated to infection [126,155,156]. An additional challenge in interpreting inflammatory biomarkers is the presence of chronic liver disease, particularly liver cirrhosis. Cirrhosis is increasingly recognized as a state of chronic systemic inflammation characterized by persistent activation of immune pathways, altered cytokine production, and immune dysregulation [157]. Elevated circulating levels of inflammatory mediators, including IL-6 and TNF-α, may persist even in the absence of acute infection, potentially affecting baseline values and postoperative kinetics of inflammatory biomarkers [157]. Furthermore, chronic liver disease is strongly associated with hepatic osteodystrophy, impaired bone metabolism, osteoporosis, and delayed fracture healing. Consequently, patients with cirrhosis undergoing orthopedic procedures may exhibit inflammatory responses and biomarker trajectories that differ substantially from those observed in otherwise healthy individuals [158,159]. These factors should be considered when interpreting perioperative inflammatory markers and assessing postoperative recovery.

Consequently, isolated biomarker measurements should never replace comprehensive clinical assessment.

Current evidence suggests that serial perioperative monitoring and interpretation of biomarker kinetics may provide greater clinical utility than isolated measurements [160]. Future prospective studies are needed to establish standardized perioperative reference trajectories and clinically validated cutoff values for orthopedic patients.

### 6.6. Conceptual Postoperative Monitoring Pathway

The interpretation of inflammatory biomarker levels after orthopedic surgery should account for the expected temporal pattern of the postoperative inflammatory response [20,21,25,26,43,73]. Based on the evidence summarized in this review, a conceptual monitoring framework may facilitate clinical interpretation of biomarker dynamics during different phases of recovery (Table 5) [20,21,22,23,24,25,73,137]. This framework is intended to support clinical decision-making and should not be considered a formal guideline.

This monitoring pathway is intended as a conceptual framework based on currently available evidence and should not be interpreted as a validated clinical guideline. It reflects the typical postoperative kinetics of inflammatory biomarkers reported in orthopedic surgery studies and reviews but has not been prospectively validated as a formal decision-support tool [20,21,22,23,24,25,26,135].

### 6.7. Future Perspectives and Personalized Perioperative Monitoring

Future research should focus on integrating inflammatory biomarkers into personalized perioperative care pathways in orthopedic surgery [130]. Rather than relying on isolated biomarker values, serial postoperative trajectories may provide more clinically meaningful information regarding normal recovery, excessive inflammatory activation, infection, thromboembolic complications, or delayed healing [27,28].

CBC-derived inflammatory indices appear particularly attractive for routine clinical practice because they are inexpensive, widely available, and obtained from standard laboratory tests [160]. However, their implementation requires prospective validation, standardized cutoff values, and procedure-specific reference ranges. This is especially important in elderly and frail orthopedic patients, in whom multimorbidity, immunosenescence, and reduced physiological reserve may substantially modify the inflammatory response [14].

In the future, combined biomarker models incorporating CRP, IL-6, PCT, D-dimers, and CBC-derived indices may improve perioperative risk stratification. Such models could be further supported by clinical variables, frailty assessment, comorbidity burden, surgical invasiveness, and postoperative recovery parameters. Artificial intelligence and machine-learning approaches may also help identify clinically relevant biomarker patterns and distinguish physiological postoperative inflammation from early complications [161,162].

Integration of inflammatory biomarker monitoring into enhanced recovery after surgery protocols may support earlier identification of high-risk patients, optimization of analgesia and mobilization strategies, rational use of antibiotics, and individualized postoperative surveillance [163]. Nevertheless, before these approaches can be routinely recommended, large prospective multicenter studies are required.

Future machine-learning models may integrate laboratory biomarkers with demographic characteristics, frailty indices, operative variables, and perioperative physiological data to improve the prediction of postoperative complications. Such multimodal approaches may facilitate earlier identification of high-risk patients and support personalized perioperative management strategies [161,162].

### 6.8. Limitations of This Review

This review has several limitations. First, due to its narrative design, no formal quantitative synthesis or meta-analysis was performed. Second, the available literature is heterogeneous across orthopedic procedures, patient populations, biomarker measurement time points, and reported clinical outcomes. Third, many studies evaluating CBC-derived inflammatory indices are retrospective and single-center in design, which limits the generalizability of their findings. Finally, the lack of standardized cutoff values remains a major limitation for the direct clinical implementation of several emerging biomarkers. Another limitation is the potential influence of chronic inflammatory comorbidities, including chronic liver disease and cirrhosis, which may alter baseline inflammatory biomarker levels, affect postoperative biomarker kinetics, and impair bone metabolism and fracture healing, thereby complicating the interpretation of perioperative inflammatory responses.

## 7. Conclusions

Orthopedic surgery induces a complex inflammatory response involving coordinated interactions between innate immunity, adaptive immune regulation, coagulation pathways, and tissue repair mechanisms. Although postoperative inflammation is essential for physiological healing and recovery, excessive or prolonged inflammatory activation may contribute to complications such as periprosthetic joint infection, thromboembolic events, delayed healing, organ dysfunction, and prolonged hospitalization.

Traditional inflammatory biomarkers, including CRP, IL-6, TNF-α, PCT, and D-dimers, remain important tools in perioperative monitoring. However, increasing evidence suggests that CBC-derived inflammatory indices, such as NLR, MLR, PLR, SII, SIRI, and AISI, may provide additional insight into the dynamic balance between systemic inflammation and immune regulation following orthopedic procedures.

Current evidence indicates that interpretation of biomarker kinetics and serial perioperative trends may be more clinically informative than isolated measurements. Furthermore, combined assessment of conventional inflammatory biomarkers and CBC-derived indices may improve postoperative risk stratification and early detection of complications.

Nevertheless, several important limitations remain unresolved, including low specificity, significant inter-individual variability, and the lack of standardized cutoff values for many novel inflammatory indices. Therefore, inflammatory biomarkers should currently be considered supportive rather than definitive diagnostic tools, and should always be interpreted in conjunction with clinical assessment, imaging, or microbiological findings, when appropriate.

From a clinical perspective, the most promising approach appears to be the combined interpretation of traditional biomarkers and CBC-derived inflammatory indices over time. Such an approach may better reflect the complexity of the postoperative inflammatory response than any single marker alone and may support individualized decision-making in orthopedic patients.

Future prospective studies are needed to establish standardized perioperative reference trajectories, validate clinically relevant cutoff values, and determine the role of inflammatory biomarkers in personalized perioperative care pathways for orthopedic patients.

## Figures and Tables

**Table 1:** Representative clinical studies evaluating inflammatory biomarkers in orthopedic surgery.

Study	Design	Population (*n*)	Procedure	Biomarkers	Key Findings
Maniar et al. [20] 2019	Prospective observational study	50	Primary TKA	IL-6, CRP	IL-6 peaked at 12 h and returned to baseline within 2 weeks, whereas CRP peaked at 48 h and remained elevated longer, suggesting superior utility of IL-6 for early postoperative monitoring.
Festa et al. [21] 2022	Systematic review and meta-analysis	7537	THA/TKA, PJI studies	NLR, MLR, PLR, PVR	CBC-derived ratios demonstrated only fair diagnostic accuracy for PJI and should not be used as standalone screening tests.
Jones et al. [22] 2024	Retrospective database study	32,868	Primary THA/TKA	NLR, MLR, PLR, SII	Elevated preoperative inflammatory ratios were associated with higher odds of postoperative complications and prolonged hospital stay.
Kürüm et al. [23] 2024	Retrospective cohort study	187	TKA	SII, SIRI	Higher SII and SIRI values were associated with increased risk of periprosthetic joint infection.
Lyubimova et al. [24] 2025	Retrospective cohort study	6036	Primary THA/TKA	SII, SIRI, AISI	Elevated systemic inflammatory indices were associated with increased risk of early periprosthetic infection following arthroplasty.
Pan et al. [25] 2024	Systematic review and meta-analysis	7212	Geriatric hip fracture	NLR, SII, RDW	Higher NLR and SII were independently associated with increased long-term mortality after hip fracture.
Yao et al. [26] 2023	Retrospective cohort study	1199	Elderly hip fracture	NLR, PLR, SII	NLR showed the best predictive performance for postoperative pneumonia and was independently associated with increased risk.
Deng et al. [19] 2024	Retrospective study	168	Revision THA/TKA	NLR, MLR, PLR, PVR	CBC-derived ratios showed only moderate diagnostic value for PJI and performed less well than combined biomarker models.
Domagalska et al. [27] 2023	Randomized controlled trial	60	Lumbar decompression and stabilization	NLR, PLR	ESPB significantly reduced postoperative NLR and PLR, opioid consumption, and pain scores compared with sham block.
Domagalska et al. [28] 2023	Randomized controlled trial	366	Total knee arthroplasty	NLR, PLR	iPACK + ACB improved pain control and functional recovery and was associated with lower postoperative NLR and PLR values.

**CBC—**complete blood count; **CRP—**C-reactive protein; **IL-6—**interleukin-6; **NLR—**neutrophil-to-lymphocyte ratio; **MLR—**monocyte-to-lymphocyte ratio; **PLR—**platelet-to-lymphocyte ratio; **PVR—**platelet-to-mean platelet volume ratio; **SII—**systemic immune-inflammation index; **SIRI—**systemic inflammatory response index; **AISI—**aggregate index of systemic inflammation; **RDW—**red cell distribution width; **PJI—**periprosthetic joint infection; **THA—**total hip arthroplasty; **TKA—**total knee arthroplasty.

**Table 2:** Kinetics and clinical characteristics of inflammatory biomarkers following orthopedic surgery. * The asterisk indicates that the expected normalization time is variable and may depend on the surgical procedure, baseline patient characteristics, postoperative complications, and timing of measurement. Serial trends are therefore generally more informative than isolated measurements.

Biomarker	Source	Time to Peak	Expected Normalization	Clinical Applicability	Limitations
CRP	Hepatocytes (IL-6 dependent)	24–48 h	7–14 days	Detection of postoperative infection, monitoring inflammatory response and antibiotic therapy	Low specificity; elevated in most inflammatory conditions
IL-6	Monocytes/macrophages, endothelial cells	6–24 h	24–72 h	Early detection of systemic inflammatory response and postoperative complications	Short half-life; limited routine availability
TNF-α	Macrophages, T lymphocytes	30–120 min	Within hours	Early-phase inflammatory response marker	Very short half-life; limited clinical applicability
PCT	Neuroendocrine cells and extra-thyroid tissues during systemic inflammation	12–24 h	2–5 days	Sepsis diagnosis and guidance of antibiotic therapy	Limited value in localized infections
D-dimer	Fibrin degradation product	1–7 days	May remain elevated for several weeks	Detection of thromboembolic complications	Low specificity in the postoperative period
NLR	CBC-derived index	24–72 h	3–7 days *	Early inflammation assessment, risk stratification, prognosis	Non-specific; influenced by surgical stress and comorbidities
MLR	CBC-derived index	Several days	Variable *	Assessment of chronic inflammation and prognosis	Limited orthopedic validation
PLR	CBC-derived index	Several days	Variable *	Evaluation of inflammatory and thrombotic activity	Affected by platelet disorders
SII	CBC-derived composite index	Several days	Variable *	Comprehensive assessment of systemic inflammation and prognosis	No universally accepted cut-off values
SIRI	CBC-derived composite index	Several days	Variable *	Prediction of adverse outcomes and mortality	Limited prospective evidence
AISI	CBC-derived composite index	Several days	Variable *	Global assessment of immune-inflammatory status	Complex interpretation; limited validation

The postoperative normalization of CBC-derived inflammatory indices varies substantially according to surgical procedure, baseline patient characteristics, postoperative complications, and timing of measurement. Therefore, serial trends are generally more informative than isolated measurements.

**Table 3:** Interpretation of perioperative kinetics of classical inflammatory biomarkers following orthopedic surgery.

Biomarker	Initial Increase	Typical Peak	Expected Normalization	Persistent Elevation May Suggest
TNF-α	Minutes after surgical trauma	30–120 min	Within hours	Severe systemic inflammation, excessive cytokine activation
IL-6	1–4 h	6–24 h	24–72 h	Cytokine storm, ongoing systemic inflammation, sepsis
CRP	12–24 h	24–48 h	7–14 days	Surgical site infection (SSI), periprosthetic joint infection (PJI), delayed recovery
PCT	4–12 h	12–24 h	2–5 days	Bacterial infection, sepsis
D-dimer	Hours	POD 1–7	May remain elevated for several weeks	Venous thromboembolism (VTE), persistent coagulation activation

**Table 4:** Clinical interpretation of CBC-derived inflammatory biomarkers in orthopedic surgery.

Biomarker	Formula	Reported Clinical Significance	Important Limitations
NLR	Neutrophils/Lymphocytes	Elevated values associated with postoperative complications, infection risk, prolonged hospitalization and poorer outcomes after arthroplasty and fracture surgery [22,25,26,99]	No universal cutoff values; affected by age, obesity, smoking, steroid therapy and chronic inflammation
MLR	Monocytes/Lymphocytes	May reflect chronic inflammatory activation and postoperative immune dysregulation [21,103,104,105,106]	Limited orthopedic validation
PLR	Platelets/Lymphocytes	Associated with inflammation-coagulation interaction and risk of postoperative complications [21,107,118]	Influenced by platelet disorders and coagulation abnormalities
SII	Platelets × Neutrophils/Lymphocytes	Associated with poorer outcomes, infection risk and frailty progression after orthopedic surgery [23,26,110]	Cutoff values vary substantially between studies
SIRI	Monocytes × Neutrophils/Lymphocytes	Associated with postoperative outcomes and infection risk [23,110,119]	Limited prospective evidence
AISI	Platelets × Monocytes × Neutrophils/Lymphocytes	Global assessment of inflammatory burden; potential predictor of postoperative complications and VTE [111,112,113,114]	Limited orthopedic evidence and complex interpretation

**Table 5:** Conceptual Postoperative Monitoring Pathway for Inflammatory Biomarkers Following Orthopedic Surgery.

Postoperative Phase	Time Period	Biomarkers of Interest	Expected Physiological Pattern	Clinical Concern If Abnormal
**Early postoperative phase**	0–48 h	IL-6, TNF-α, CRP, WBC count, NLR	Rapid increase due to surgical trauma and activation of innate immunity	Excessive elevation, hemodynamic instability, or disproportionate inflammatory response may warrant closer monitoring
**Intermediate postoperative phase**	Postoperative days 3–7	CRP, PCT, NLR, PLR, SII, SIRI	Gradual decline of inflammatory markers after reaching peak values	Persistent elevation or secondary increase may suggest surgical site infection (SSI), periprosthetic joint infection (PJI), or delayed recovery
**Late postoperative phase**	>7 days	CRP, ESR, D-dimer, CBC-derived indices	Progressive normalization toward baseline values	Persistent abnormalities may indicate chronic inflammation, infection, thromboembolic complications, impaired healing, or nonunion
**Long-term follow-up**	Weeks to months	CRP, ESR, selected CBC-derived indices	Stable normalization in uncomplicated recovery	Ongoing elevation may require further diagnostic evaluation for implant-related complications or chronic inflammatory conditions

**Abbreviations:** CBC—complete blood count; CRP—C-reactive protein; ESR—erythrocyte sedimentation rate; IL-6—interleukin-6; NLR—neutrophil-to-lymphocyte ratio; PCT—procalcitonin; PLR—platelet-to-lymphocyte ratio; SII—systemic immune-inflammation index; SIRI—systemic inflammatory response index; SSI—surgical site infection; PJI—periprosthetic joint infection.

## Data Availability

No new data were created or analyzed in this study.

## Supplementary Materials

The following supporting information can be downloaded at: https://www.mdpi.com/article/10.3390/jcm15145399/s1, Figure S1: Prisma-style flow diagram of study identification, screening, eligibility assessment and inclusion for the present narrative review; Table S1: Representative study-specific cut-off values reported for CBC-derived inflammatory biomarkers in orthopedic surgery [19,23,24,26].
