# Supplementary material for: Inflammatory Biomarkers Following Orthopedic Surgery: Current Evidence, Clinical Applications, and Future Perspectives—A Narrative Review
Source: J Clin Med. 2026 Jul 9;15(14):5399. doi: 10.3390/jcm15145399 (PMC13411423; doi:10.3390/jcm15145399)

Figure S1. PRISMA-style flow diagram of study identification, screening, eligibility assessment, and inclusion for the present narrative review

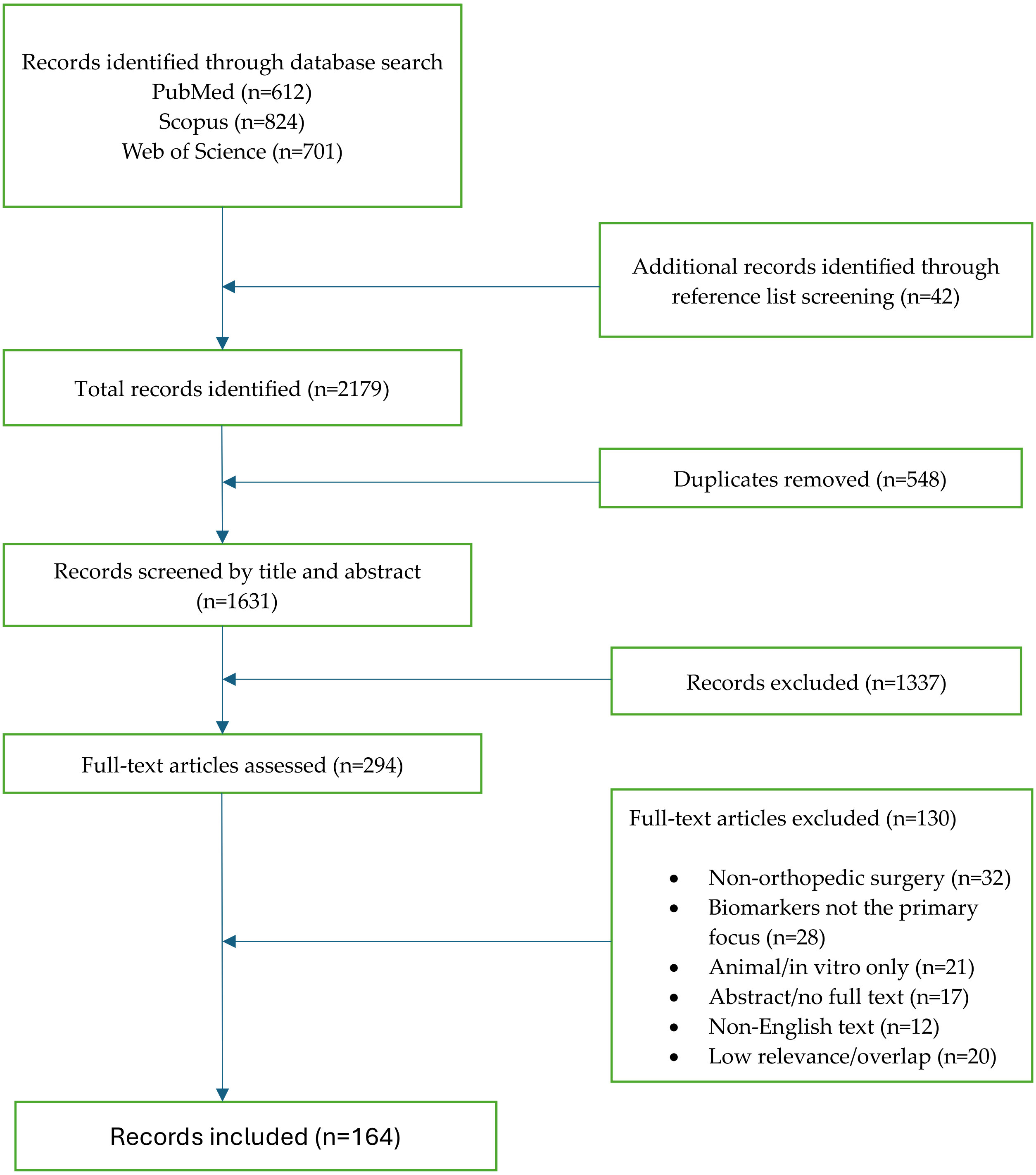

Supplement: Supplementary file 1 [file jcm-15-05399-s001.zip › jcm-4365677-supplementary.pdf]
